# Enhancing Hospital Services: Achieving High Quality Under Resource Constraints

**DOI:** 10.1177/11786329251331311

**Published:** 2025-04-11

**Authors:** Mohammad Ali Beheshtinia, Masood Fathi, Morteza Ghobakhloo, Muhammad Faraz Mubarak

**Affiliations:** 1 Industrial Engineering Department, Faculty of Engineering, Semnan University, Iran; 2 Division of Intelligent Production Systems, School of Engineering Science, University of Skövde, Sweden; 3 Division of Industrial Engineering and Management, Uppsala University, Sweden; 4 Faculty of Computer Science, Dalhousie University, Halifax, NS, Canada

**Keywords:** quality management, hospital services, quality function deployment, house of quality, service quality

## Abstract

**Objectives:**

This research aims to enhance the quality of hospital services by utilizing Quality Function Deployment (QFD) with a novel Multi-Dimensional House of Quality (MD-HOQ) approach. This method integrates Service Quality (SERVQUAL) analysis and considers resource constraints, such as financial and workforce limitations, to select and prioritize technical requirements effectively.

**Methods:**

The proposed MD-HOQ approach was applied to a private hospital in Tehran, Iran. Data were gathered from a sample of 8 experts and a sample of 386 patients, using 2 in-depth interviews and 4 questionnaires. The process included identifying hospital sections and determining their importance using the Analytic Hierarchy Process. Patients’ needs in each section were then identified and weighted through SERVQUAL analysis. Subsequently, technical requirements to meet these needs were listed and weighted using MD-HOQ. A mathematical model was employed to determine the optimal set of technical requirements under resource constraints.

**Results:**

Application of the MD-HOQ approach resulted in the identification of 50 patient needs across 5 hospital sections. Additionally, 40 technical requirements were identified. The highest implementation priorities were assigned to “training practitioners and nurses,” “improving the staff’s sense of responsibility,” and “using experienced specialists, physicians, and surgeons.”

**Conclusions:**

The integrated QFD approach, utilizing MD-HOQ and SERVQUAL analysis, provides a comprehensive framework for hospital managers to prioritize technical requirements effectively. By considering resource constraints and the gap between patient expectations and perceptions, this method ensures that resources are allocated to the most impactful technical requirements, leading to improved patient satisfaction and better overall hospital service quality. This approach not only enhances the quality of hospital services but also ensures efficient utilization of resources, ultimately benefiting patient satisfaction.

## Introduction

Healthcare services play a critical role in determining the living standards of any society, as their effectiveness significantly impacts both the quality and length of life within a community.^1,2^ With the rapidly growing demand for healthcare services, there is an increasing need for practical solutions to enhance their quality.^3^ Hospitals serve as the primary health service providers for citizens.^4^

Improving the quality of hospital services has profound benefits, including lower mortality rates, shorter hospital stays, better recovery outcomes, fewer medical errors, and reduced legal complaints against hospitals.^5-7^ Moreover, it enhances patient safety, satisfaction, trust in healthcare staff, and the overall reputation of the hospital.^7-9^ Conversely, poor-quality hospital services can negatively affect human health, the economy, and society,^10-13^ These impacts include higher mortality rates due to misdiagnosis, ineffective treatments, or delays in services, as well as the exacerbation of illnesses caused by inadequate or improper care. Economically, poor quality leads to increased treatment costs due to repeated or prolonged care, greater financial burdens on families and the healthcare system, and reduced societal productivity caused by absenteeism among staff and patients. Socially, it results in a decline in the quality of life for patients and their families. Legally and ethically, it increases complaints and lawsuits against hospitals and healthcare staff. Additionally, poor-quality services create secondary impacts on the healthcare system, such as greater pressure on hospitals and medical resources due to the higher frequency of repeat visits. Highlighting these effects shows the critical need for improving hospital service quality to mitigate these risks and enhance healthcare outcomes.

According to the latest available statistics up to 2022, Iran has 1054 hospitals with a total of 159 697 active inpatient beds.^14^ Of these, 905 hospitals operate as general hospitals, with a total of 129 884 active beds.^15^ In addition to hospitals, a significant number of other healthcare facilities, such as clinics, polyclinics, and health centers, are operational across the country. One of the most significant healthcare reforms in Iran, the “Health Sector Evolution Plan,” was initiated in 2014.^16^ Among its primary goals is enhancing the quality of hospital services. While the reform has led to positive changes, such as a reduction in in-hospital mortality rates, challenges remain. For example, higher rates of readmission within 30 days post-reform highlight areas requiring further improvement in care quality.^17^

This research aims to propose a hybrid method by integrating the Multi-Dimensional House of Quality (MD-HOQ), Analytic Hierarchy Process (AHP), and SERVice QUALity (SERVQUAL) methods to enhance the quality of hospital services while considering resource constraints such as financial and human resources. To evaluate the performance of the proposed method, it was implemented in a real-life private hospital in Tehran, Iran.

The House of Quality (HOQ) is a 2-dimensional matrix that serves as the core of the Quality Function Deployment (QFD) method. QFD is one of the widely used tools for enhancing service quality in healthcare that captures and translates customer (ie, patient) needs into technical requirements. In HOQ matrix, customer needs are listed in the rows, while technical requirements are listed in the columns. Each technical requirement is weighted based on its impact on customer satisfaction, and implementing these requirements leads to quality enhancement in the organization.^18^

While several studies have utilized QFD to enhance service quality in hospitals and healthcare centers, none have adequately addressed the constraints posed by limited resources, including financial and workforce, in the selection and implementation of technical requirements. Additionally, these studies typically consider only a 2-dimensional HOQ matrix, which limits their application in real-world scenarios.

The main objectives of this study include adopting a cross-sectoral perspective to enhance hospital service quality. Traditional HOQ methods are often unsuitable for hospitals due to their multi-dimensional nature, as each section has varying levels of importance. Implementing QFD for each section independently can lead to diverse plans and inefficiencies. The proposed MD-HOQ addresses this by applying QFD across multiple hospital sections to create a comprehensive and unified list of technical requirements, ensuring efficient resource utilization and consistency. The final weight of each technical requirement reflects its impact on patient satisfaction across all sections rather than focusing on a single section. Additionally, the MD-HOQ incorporates the relative importance of different hospital sections when calculating the weight of technical requirements. Using AHP ensures that technical requirements that satisfy the needs of more critical sections are given higher priority. This study also addresses patients’ critical needs by enhancing the traditional QFD approach. Beyond the usual parameters—such as the number of patients’ needs addressed, the intensity of the impact, and the importance of the needs satisfied—this research introduces the gap between patient expectations and perceptions, measured through SERVQUAL analysis. A higher gap indicates greater dissatisfaction, necessitating focused attention. Lastly, the study considers resource constraints, recognizing that organizational resources like financial and workforce capacities are limited and may not suffice to implement all identified technical requirements. Therefore, the proposed method prioritizes technical requirements that maximize patient satisfaction while accounting for resource consumption and the relative importance of each requirement, with financial and workforce limitations determined through expert opinions.

The main research question of this study can be stated as follows: What is the best set of technical requirements to enhance the quality of hospital services under resource constraints, including financial and workforce constraints?

To answer this, the study addresses the following sub-questions:

- What is the degree of importance of each section at the considered hospital?
- What are the patients’ needs in each section?
- What is the importance degree of each patient need?
- What is the gap between patients’ expectations and perceptions for each need?
- What is the unique list of technical requirements for satisfying patient needs across different sections?
- What are the relationships between the identified technical requirements and patient needs in different sections?
- What are the implementation priorities and costs of the identified technical requirements?

The rest of the paper is ordered as follows: The previous research is reviewed, and the research gaps are identified in Section 2. Section 3 outlines the methodology and proposed approach. Section 4 discusses the results of implementing the proposed approach in a private hospital. Finally, Section 5 provides the conclusions.

## Literature Review

This section reviews the most relevant studies that applied QFD to enhance the quality of healthcare services.

Camgöz-Akdağ et al^19^ combined SERVQUAL and QFD to determine success factors for quality improvement in the healthcare industry. Azadi and Farzipoor Saen^20^ employed QFD and Data Envelopment Analysis (DEA) to enhance the quality of healthcare services, using imprecise data within the context of imprecise enhanced Russell graph measures to consider criteria such as cost and ease of implementation. Using a combination of QFD and SERVQUAL, Chou et al^21^ provided a prioritization list for creating a friendly hospital environment and identifying service gaps. Lee et al^3^ used QFD and fuzzy numbers to enhance healthcare service quality, considering 7 customer needs and 17 technical requirements in their QFD.

Gao and Zhang^22^ combined QFD and SERVQUAL to estimate hidden costs of quality caused by patient dissatisfaction in healthcare. Wood et al^23^ used QFD for green hospital design in Malaysia, considering 18 demand qualities in the rows of the HOQ matrix and 13 quality design elements in the columns. Dehe and Bamford^24^ used QFD to enhance strategic operations in healthcare organizations in the United Kingdom, identifying 15 service and building design attributes to satisfy 10 customer needs. Jonny and Zagloed^25^ discussed quality enhancement in the Indonesian healthcare system by merging QFD, Kano’s model, and the balanced scorecard, focusing on maximizing natural light usage, ventilation, water-efficient equipment, and environmentally friendly materials. Priyono and Yulita^26^ integrated QFD and the Kano model to enhance the quality in the front office of a hospital in Indonesia, identifying 14 patient needs categorized into “attractive needs,” “one-dimensional needs,” and “must-be needs.”

Raziei et al^27^ employed group decision-making, fuzzy theory, SERVQUAL, and QFD to enhance service quality in a public hospital, considering 25 customer needs and proposing 23 technical requirements. Maalej et al^28^ merged QFD, risk analysis, and fuzzy numbers to enhance the quality of healthcare services, considering 5 patient needs and 7 technical requirements, and calculating a risk priority number for each technical requirement. Hasibuan et al^29^ used QFD to enhance the quality of a public general hospital, considering 21 patient needs and discussing their importance and gaps between patient interests and hospital service levels. Susanto and Wurjaningrum^30^ combined service blueprint analysis and QFD to determine business process services in the inpatient room of a Hospital in Indonesia, identifying 10 customer needs and 10 technical requirements. Joshi and Bhargava^31^ discussed the integration of Green QFD and waste management in healthcare centers, comparing the results of QFD and Green QFD, and implementing their method in both a private and a state hospital.

Mathews et al^32^ implemented QFD to prioritize healthcare solutions for pressure ulcers, analyzing the development costs, usage costs, and development time for each technical requirement. Altuntas and Kansu^33^ merged SERVQUAL, QFD, and failure modes and effects analysis to improve service quality and eliminate potential failures during service delivery in a public hospital in Turkey.

Tortorella et al^34^ discussed the implementation and integration of healthcare 4.0 in hospitals using QFD, considering healthcare value chain problems as rows in the QFD matrix and healthcare 4.0 digital applications as columns, implementing their method in hospitals in Brazil and India. Lu et al^35^ combined the Kano model and QFD to design a pre-hospital emergency system, showing the procedures and their ranks. Junior et al^36^ integrated QFD, fuzzy theory, and SERVQUAL analysis to create a quality planning tool for surgical centers, implementing the model in a public hospital in Brazil and considering the most relevant deficiencies as QFD matrix entries. Khan et al^37^ integrated fuzzy theory, QFD, and the full consistency method to evaluate strategies for enhancing healthcare sector resilience in Pakistan during the COVID-19 pandemic. They used QFD to establish relationships between resilient attributes and strategies. Nie et al^38^ employed QFD to improve healthcare service quality, using the best-worst method to determine customer needs and interactive and multi-criteria decision-making to determine technical requirement weights.

Raj and Samuel^39^ used QFD to identify barriers to healthcare waste management in India, showing the elements that affect waste management. Gavahi et al^40^ combined QFD and SERVQUAL to enhance service quality in radiology centers of public hospitals in Tehran, Iran, categorizing patient requirements into the 5 dimensions of SERVQUAL. Chowdhury et al^41^ used QFD to determine resilience strategies to improve vaccine operations and distribution performance during the COVID-19 pandemic, using fuzzy set qualitative comparative analysis to prioritize strategies.

### Review Summary and Key Insights

The literature review reveals that while some studies have utilized pure QFD, the trend in recent research is to merge QFD with other techniques, such as SERVQUAL and various multi-criteria decision-making (MCDM) methods. These combinations aim to enhance the robustness and applicability of QFD in improving healthcare service quality. The review also shows that none of the studies have implemented an MD-HOQ. The traditional 2-dimensional HOQ limits the application of QFD in complex organizational structures, such as hospitals with multiple departments. Additionally, no research has considered resource constraints, including financial and workforce constraints, when selecting and implementing technical requirements. This omission can lead to impractical solutions that are not feasible in real-world settings where financial resources are limited.

To address these gaps, this research proposes a new approach with several main contributions. First, implementing an MD-HOQ allows it to be applied across different sections of hospitals, facilitating the creation of a comprehensive and unified list of technical requirements that address the needs of multiple departments. Second, by integrating the MD-HOQ with SERVQUAL and AHP, this research introduces a more precise method for determining the weights of technical requirements while considering resource constraints. Finally, the proposed solution is validated through implementation in a real-life hospital, demonstrating its practicality and effectiveness in enhancing hospital service quality under resource constraints.

## Methodology

This study presents a modified QFD approach with an overarching view, named MD-HOQ, integrated with SERVQUAL analysis to improve hospital service quality under resource constraints. In the proposed hybrid method, based on the patients’ needs, the technical requirements to satisfy them are identified. Based on the impact of technical requirements on patients’ needs (considering the importance degrees, perceptions, and expectations of the affected patients’ needs), the weights of the technical requirements are determined. Finally, the optimum set of selected technical requirements, based on resource constraints, is identified. This section describes the tools used, the data-gathering process, and the research steps. Section 4, in contrast, presents the results obtained from implementing these research steps in a real-life hospital.

### MD-HOQ

**Figure 1.**
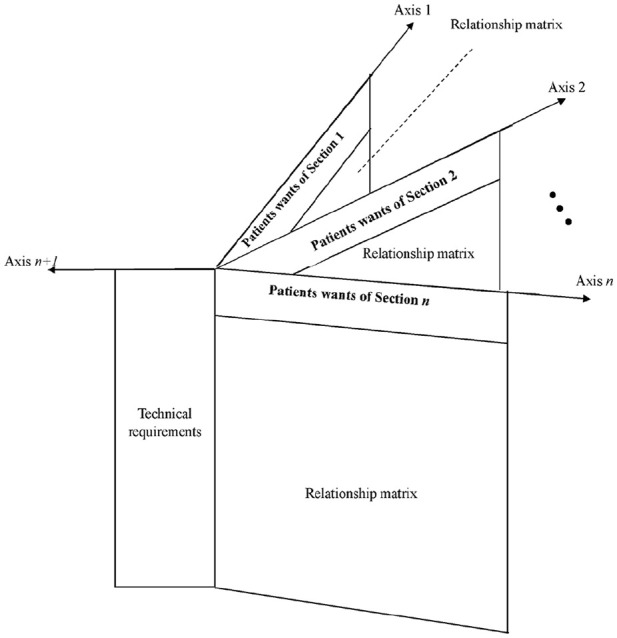
The structure of MD-HOQ.

Although the traditional HOQ has 2 dimensions (1 dimension for technical requirements and 1 dimension for patients’ needs), the proposed MD-HOQ has multiple dimensions. If MD-HOQ is implemented in *n* different sections, it has (n + 1) dimensions (1 dimension for technical requirements and *n* dimensions for patients’ needs in each section). Figure 1 shows the structure of the proposed MD-HOQ.

### Date Gathering

In this study, 2 in-depth interviews and 4 types of questionnaires were employed to collect the required data. Data collection took place over a period of 5 months (from February 1, 2024, to June 30, 2024).

#### The Used Samples

Two groups, experts and patients, were included as participants in the interviews and questionnaires. Detailed information about these samples is provided below:

*Patient Sample:* A sample of 386 patients was randomly selected, and the sample size was determined using the Cochran formula with a 5% margin of error39. The hospital had a total of 16 491 visits across the selected sections (surgery, emergency, CCU, physiotherapy, and pediatrics) during the 5-month study period. Stratified sampling was applied to ensure representative patient sampling. Detailed information on the patient sample is presented in Table 1.

**Table 1.** Detail information about sample of patients.

Sample of patients				Sample of experts			
Category	Total patients	Percentage of total population (%)	Sample size	No.	Job	Academic degree	Experiences (years)
Gender				1	Hospital manager	Doctor of Medicine	14
Male	7756	47.03	182	2	Head of surgery section	Fellowship	12
Female	8735	52.97	204	3	Head of the emergency section	Fellowship	11
Sections				4	Head of CCU section	Fellowship	12
Surgery	1868	11.33	44	5	Head of the physiotherapy section	Fellowship	14
Emergency	5450	33.05	128	6	Head of pediatrics section	Fellowship	18
CCU	1918	11.63	45	7	Head nurse of the surgery section	M.Sc. in Nursing	11
Physiotherapy	2878	17.45	67	8	Head nurse of the emergency section	M.Sc. in Nursing	12
Pediatrics	4377	26.54	102				
Level of care							
Primary care	9476	57.46	222				
Secondary care	4474	27.13	105				
Tertiary care	2541	15.41	59				

*Expert Sample:* The expert sample included 8 individuals selected through purposeful sampling based on the following criteria: (1) A minimum academic qualification of MSc or higher, (2) Over 10 years of professional experience in healthcare, and (3) Adequate knowledge of the selected sections or relevant management scopes.

Detailed information about the expert sample is also presented in Table 1.

#### The Interviews and Questionnaires

Patients’ needs were identified using 2 methods: recorded patient comments from the hospital’s website and in-depth interviews with patients, referred to as the first interview. To complement this, a second in-depth interview was conducted with experts to determine the technical requirements necessary to address patients’ needs and to assess the cost and workforce required. In addition to the interviews, 4 questionnaires were utilized to gather comprehensive data for the study. The first questionnaire employed pairwise comparison matrices to evaluate the importance of various hospital sections, while the second questionnaire focused on assessing the significance of patients’ needs. The third questionnaire measured patients’ expectations and perceptions regarding each identified need, and the fourth questionnaire investigated the relationships between technical requirements and patients’ needs. The respondents for the first and fourth questionnaires were selected from the expert sample, while the second and third questionnaires were completed by the patient sample. Table 2 presents the linguistic terms used in the questionnaires, their corresponding numerical values, and the respondents for each questionnaire.

**Table 2.** Linguistic terms used in the questionnaires.

	The first questionnaire (Pairwise comparisons)		The second questionnaire (Importance degree)		The third questionnaire (SERVQUAL)		The fourth questionnaire (Relationship matrix)	
Subject	Value	Linguistic term	Value	Linguistic term	Value	Linguistic term	Linguistic term	Value
	1	Equally strong	1	Very Low	1	Completely disagree	Weak (△)	1
	3	Moderately strong	2	Low	2	Disagree	Moderate (〇)	3
	5	Strong	3	Medium	3	No Difference	Strong (●)	9
	7	Very strong	4	High	4	Agree	No relationship	0
	9	Extremely strong	5	Very High	5	Completely agree		
Respondent	Sample of experts		Sample of patients		Sample of patients		Sample of experts	
Number of questions	33		50		100		2000	

The reliability of the first questionnaire is validated by an average inconsistency rate of 0.02, while the reliability of the second, third, and fourth questionnaires is validated by Cronbach’s alpha values of .88, .81, and .79, respectively. All the questionnaires are standard, and their validity has been confirmed.^42,43^

### Research Steps

**Figure 2.**
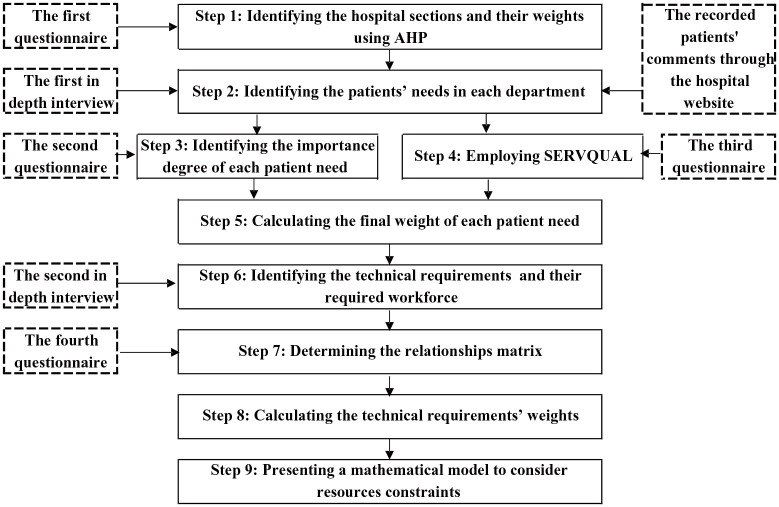
An overview of the research steps.

This section describes the research steps taken to answer the research questions. Figure 2 provides an overview of these steps.

*Step 1: Identifying the hospital sections and their weights*

In this step, the hospital sections with different patients (customers) are identified, and the degree of importance of each section is determined.

Due to budget limitations, not all hospital sections could be included in the quality enhancement procedure. Therefore, initial filtering was applied based on the following 3 parameters: (1) The number of patients (as sections with higher patient numbers have the greatest impact on overall patient perception of hospital services), (2) Income generation by the section (since the hospital is private, sections with higher income potential are prioritized for quality improvements), and (3) Section criticality (sections identified as critical in terms of patient dissatisfaction, such as those with a high number of complaints or issues from previous feedback, which could harm the hospital’s reputation).

Based on these criteria, 5 sections—surgery, emergency, cardiac care unit (CCU), physiotherapy, and pediatrics—were selected as alternatives. The importance degrees of these sections are calculated based on the above criteria using AHP method. Readers interested in learning more about AHP are referred to.^44,45^

If there are *n* criteria and *m* alternatives, the procedures of the AHP method are as follows:

- Create a pairwise comparison matrix for all the criteria (*P*). This matrix is an *n× n* matrix. The arrays in this matrix show the privilege of criteria against each other.

(1) $$P={\left[p_{kj}\right]}_{n\times n}=\;\;\left[\begin{array}{ccccc} p_{11} & p_{12} & p_{13} & \ldots & p_{1n}\\ p_{21} & p_{22} & p_{23} & \ldots & p_{12}\\ p_{31} & p_{32} & p_{33} & \ldots & a_{13}\\ \ldots & \ldots & \ldots & \cdots & \ldots \\ \ldots & \ldots & \ldots & \ldots & \ldots \\ p_{n1} & p_{n2} & p_{n3} & \cdots & p_{nn} \end{array}\right]$$

- Normalize matrix *P* using equation (2), and name it matrix *NORM*,

(2) $$NORM={\left[norm_{kj}\right]}_{n\times n},\;norm_{kj}=p_{kj}∕\overset{n}{\underset{k=1}{\sum }}p_{kj}$$

- Calculate the criteria weights employing equation (3). In this equation, $WC_{j}$ represents the weight of criterion *j* (*j* *=*1, 2,*. . ., n*).

(3) $$WC_{j}=\overset{n}{\underset{k=1}{\sum }}\frac{norm_{kj}}{n}\;$$

- For each criterion *j*, obtain an *m× m* pairwise comparison matrix to compare alternatives and obtain the weight of alternatives for each criterion. Let $WA_{i}^{j}$ be the weight of alternative *i* in criterion *j*.

Obtain the total weight (importance degree) of each alternative *i* (*DW_i_*) using equation (4).

(4) $$DW_{i}=\overset{n}{\underset{j=1}{\sum }}WC_{j}\times WA_{i}^{j}\;i=1,\;2,\ldots ,m$$

The pairwise comparison matrices are obtained using the pairwise comparison questionnaire. In this questionnaire, the elements (criteria or alternatives) are compared. In each comparison, the decision-makers have several choices to indicate the preference of one element over another. Each response is a human judgment expressed using a linguistic term^46^

*Step 2: Identifying the patients’ needs in each section*

In this step, patients’ needs in each section were determined through in-depth interviews with patients and by analyzing records of their comments submitted via the hospital’s website. These identified needs were then reviewed by experts to eliminate duplicates and overlaps, ensuring a concise and accurate list of requirements.

*Step 3: Identifying the importance degree of each patient*’*s needs*

The degree of importance of the patients’ needs is determined using the customer importance degree questionnaire (ie, the second questionnaire). In this questionnaire, the customers rate the importance of each identified need based on the Likert scale shown in Table 2. The patients’ needs with higher importance degrees should be given more attention by the managers.

*Step 4: Employing SERVQUAL*

To determine the final weight of each patient’s needs, SERVQUAL analysis is used to identify the gap between expected service and experienced (perceived) service. For this purpose, the patients’ perceptions and expectations are assessed using the SERVQUAL questionnaire. In this questionnaire, patients rate their perceptions and expectations for each identified need based on the Likert scale shown in Table 2. Finally, an index named improvement ratio (*IR_i_*) is defined and calculated using equation (5), where $P_{i}^{d}$ and $E_{i}^{d}\;$ are the patient perceptions and expectations of the ith patient’s need in the *d*th section, respectively. The greater the gap between the expectation and perception of a patient’s need *i*, the higher the improvement ratio, indicating that this need requires more attention from hospital managers. Improving the quality provided (patient perception) will reduce the gap with patient expectations, leading to higher patient satisfaction.

(5) $$IR_{i}^{d}\;=\;E_{i}^{d}/P_{i}^{d}$$

*Step 5: Calculating the final weight of each patient*’*s need*

To determine the final weight of patients’ needs, both the importance degree and improvement ratio are considered. The absolute weight ( $AW_{i}^{d}$ ) and the relative weight ( $RW_{i}^{d}$ ) of the ith patient’s need in the *d*th section are obtained using equations (6) and (7), respectively.

(6) $$AW_{i}^{d}\;=\;I_{i}^{d}\;\times \;\;{\mathrm{IR}}_{i}=I_{i}^{d}\;\times \;\;(E_{i}^{d}/P_{i}^{d})$$

(7) $$RW_{i}^{d}=AW_{i}^{d}/\overset{n^{d}}{\underset{i=1}{\sum }}AW_{i}^{d}$$

In equation (7), $n^{d}\;$ is the number of patients’ needs in the *d*th section. The greater the weight of the patient’s need, the higher its priority compared with other needs. Equation (6) shows that the priority of each patient’s need depends not only on its importance but also on the hospital’s delay in satisfying this need.

*Step 6: Identifying the technical requirements*

This step identifies the technical requirements for satisfying the patients’ needs using in-depth interviews with experts.

*Step 7: Determining the relationship matrix*

In this step, the relationship between each patient’s needs and each technical requirement is determined. Table 2 shows the various relationship types and the corresponding number for each type. In this case, 5 relationship matrices should be obtained (a relationship matrix for each section).

*Step 8: Calculating the technical requirements’ weights*

If QFD is implemented in each section separately, then different lists of technical requirements will be obtained, each with different weights (importance degrees) for each technical requirement. Some technical requirements only affect the patients’ needs in a single section, while others may affect multiple sections simultaneously. Therefore, the technical requirements that affect multiple sections could receive higher weight, even if they might not have a high weight in each individual section.

For better clarification, consider 2 technical requirements (ie, *A* and *B*), which only affect one section, where *A* has a higher weight than *B*. In this case, *A* gets a higher priority for implementation and will be prioritized. However, assuming technical requirement *B* affects the patients’ needs in multiple sections, it may have more benefits for the hospital. Thus, it should get a higher implementation priority, even if it has a lower weight in one specific section. To have an overarching view in MD-HOQ, the weight of each technical requirement is calculated using equations (8) and (9).

(8) $$ATW_{j}=\overset{F}{\underset{d=1}{\sum }}\overset{n^{d}}{\underset{i=1}{\sum }}DW_{d}\times r_{ij}^{d}\times RW_{i}^{d}$$

(9) $$RTW_{j}=\frac{ATW_{j}^{d}}{\sum_{j=1}^{Z}ATW_{j}^{d}}$$

In these equations, *F*, *Z*, $ATW_{j}$ , $RTW_{j}$ , $DW_{d}$ , $r_{ij}^{d}$ represent the number of sections, the number of technical requirements, the absolute total weight of technical requirement *j*, its relative total weight, the weight of the section obtained in Step 1, and the relation of the *j*th technical requirement and the *i*th patient need in the *d*th section, respectively.

The weight of each technical requirement shows its total effect on patients’ satisfaction. In other words, the weight of each technical requirement shows its benefit (utility) and implementation priority for the hospital. The higher weight of each technical requirement indicates a higher implementation priority for the hospital. The weight of technical requirements depends on the following parameters:

- The number of patient needs affected by a technical requirement: As the number of patient needs affected by a technical requirement increases, the weight of that technical requirement also increases.
- The importance of patient needs affected by a technical requirement: If a technical requirement addresses patient needs with higher importance, the weight of that technical requirement increases.
- The significance of hospital sections affected by a technical requirement: If a technical requirement impacts patient needs in more critical sections, the weight of that technical requirement increases.
- The intensity of a technical requirement’s effect on patient needs: If a technical requirement has a substantial impact on patient needs (●), its weight is higher compared to a technical requirement with a weaker effect (△).

*Step 9: Considering the resource constraints*

While it is desirable to implement all the technical requirements, resource constraints often prevent this. Therefore, the hospital manager must select a subset of the technical requirements based on the available resources. This study proposes a mathematical formulation, presented in equations (10)-(12), to determine the optimal set of technical requirements while adhering to resource constraints, including financial and workforce, under the following assumptions.

- Implementing each technical requirement *j* incurs a cost denoted by *C_j._*
- The hospital’s total budget for implementing the technical requirements is predetermined and represented by *B*.
- Each technical requirement *j* needs $H_{j}$ Man-hour of the executive team (in the considered case, the executive team is composed of 5 staff from the administrative and financial section)
- The hospital’s total available workforce of the executive team is predetermined and represented by *AWF*.
- The benefit of each technical requirement *j* for the hospital ( $RTW_{j}$ ) is determined using the MD-HOQ, as described by equation (9).

The objective is to maximize the total benefit (utility) of implementing the technical requirements while staying within the resource’s constraints.

(10) $$Max\;Z=\overset{z}{\underset{j=1}{\sum }}RTW_{j}\times X_{j}$$

 Subject to:

(11) $$\overset{z}{\underset{j=1}{\sum }}C_{j}\times X_{j}\leq B$$

(12) $$\overset{z}{\underset{j=1}{\sum }}H_{j}\times X_{j}\leq \mathrm{AWF}$$

Where *X_j_* is a binary decision variable $\left(X_{j}\in \left\{0,1\right\}\right)$ . It takes the value 1 if the *j-*th technical requirement is selected for implementation, and 0 otherwise.

## Results

To examine the proposed method, it was implemented in a case study at a 300-bed private hospital located in Tehran, Iran. Constructed in 1998, the hospital spans 12 stories and covers a total area of 4300 square meters. The high population of Tehran city (more than 9 million citizens) and the resulting high demand for hospital services prompted the selection of a hospital in this city as the case study. Additional reasons include its private status (since competition among private hospitals to attract patients is intense, enhancing service quality is critical for survival) and its good accessibility. The results of applying the research steps (outlined in Section 3.3 and illustrated in Figure 2) to this hospital are detailed in this section.

### Hospital Sections and Their Importance Degrees

In the first step, the 5 most important and critical sections were selected: Surgery (I), Emergency (II), Cardiac Care Unit (CCU) (III), Physiotherapy (IV), and Pediatrics (V). Their relative importance was then determined using the AHP method, based on 3 criteria: number of patients, criticality, and generated income. After implementing the AHP method, the importance degrees for sections I to V were found to be 0.486, 0.252, 0.116, 0.063, and 0.083, respectively.

### Patient’s Needs in Each Section

In this step, the patients’ needs in each section were identified. It is important to note that the needs of patients in one section may differ from those in other sections. Ultimately, 50 patients’ needs were identified across the 5 hospital sections, with 10 high-priority needs for each section. These are detailed in Table 3 (*CW*^*d*^_*i*_ denotes the *i*th customer need in the *d*th section).

**Table 3.** The identified patient needs and their notations.

Notation	Want	Notation	Want
	**Surgery (Section I)**	*CW* ^III^_6_	Availability of modern and adequate equipment for the CCU
*CW* ^I^_1_	Guarantee for improving the patient’s condition after surgery	*CW* ^*^III^*^_7_	Suitable environment for visitors
*CW* ^I^_2_	Sufficient and modern surgical equipment	*CW* ^III^_8_	Monitoring the patient’s condition 24 × 7
*CW* ^*^I^*^_3_	Take measures to reduce the stress of the visitors	*CW* ^*^III^*^_9_	Appropriate response to the caregivers
*CW* ^I^_4_	The existence of a friendly environment	*CW* ^*^III^*^_10_	Special attention to patients with no consciousness
*CW* ^*^I^*^_5_	Low waiting time for surgery		**Physiotherapy (Section IV)**
*CW* ^*^I^*^_6_	A quiet environment without sound pollution	*CW* ^IV^_1_	Sufficient and modern physiotherapy equipment
*CW* ^^*I*^^_7_	Possibility of presenting a patient’s health status to their caregivers at any time	*CW* ^IV^_2_	Proper behavior of experts and employees
*CW* ^*^I^*^_8_	The presence of adequate staff, including nurses and practitioners, in the section	*CW* ^*^IV^*^_3_	The safety of devices and equipment
*CW* ^I^_9_	Accountability and guidance for the visitors	*CW* ^*^IV^*^_4_	The familiarity of patients with how the equipment works
*CW* ^I^_10_	Attending the patient after surgery	*CW* ^*^IV^*^_5_	Sufficient broadness of the area concerning the number of visitors
	**Emergency (Section II)**	*CW* ^IV^_6_	Well-organized environment and equipment
*CW* ^*^II^*^_1_	Safe transfer of patients to the emergency section	*CW* ^*^IV^*^_7_	Helping the patients
*CW* ^*^II^*^_2_	Quick transfer of patient	*CW* ^*^IV^*^_8_	Providing enough information to visitors
*CW* ^II^_3_	Timely and appropriate treatment	*CW* ^*^IV^*^_9_	The availability of facilities to meet the basic needs of patients
*CW* ^*^II^*^_4_	Patients not being infected with other diseases	*CW* ^*^IV^*^_10_	Flexible and open space for activities
*CW* ^*^II^*^_5_	Giving enough attention to patients		**Pediatric (Section V)**
*CW* ^*^II^*^_6_	Presence of sufficient medicines and equipment for emergencies	*CW* ^^*V*^^_1_	Reduce children’s fear and stress
*CW* ^II^_7_	No congestion in the section	*CW* ^*^V^*^_2_	Healthy and appealing food for children
*CW* ^*^II^*^_8_	Improving the visitors’ morale	*CW* ^*^V^*^_3_	Suitability of the environment for the children
*CW* ^*^II^*^_9_	Suitable environmental elements according to emergency conditions	*CW* ^*^V^*^_4_	Good behavior of practitioners and nurses with children
*CW* ^II^_10_	Having professional practitioners and nurses in all working shifts	*CW* ^V^_5_	Provide enough information to children’s caregivers
	**CCU (Section III)**	*CW* ^V^_6_	Improving children’s morale
*CW* ^III^_1_	Patient privacy	*CW* ^*^V^*^_7_	Safe equipment according to children’s physical conditions
*CW* ^^*III*^^_2_	Having enough beds and low waiting time for hospitalization	*CW* ^*^V^*^_8_	Fast treatment regarding children’s intolerance
*CW* ^*^III^*^_3_	High-quality and healthy food	*CW* ^*^V^*^_9_	Special attention to the children’s health
*CW* ^*^III^*^_4_	Food variety	*CW* ^*^V^*^_10_	Suitable environment for the patient’s caregivers
*CW* ^*^III^*^_5_	Clean environment		

### Identifying the Importance Degree of Each Patient’s Needs

Table 4 presents the importance degrees obtained from the second questionnaire. The results indicate that in sections I to V, the highest importance degrees are assigned to the following patient needs: “The presence of adequate staff, including nurses and practitioners in the section,” “Giving enough attention to patients,” “Special attention to patients with no consciousness,” “The availability of facilities to meet the basic needs of patients,” and “Special attention to children’s health,” respectively.

**Table 4.** The importance degree, perceptions, expectations, and weights of patients’ needs.

Want	Section	Importance degree	Patient expectation	Patient perception	Improvement ratio	Absolute weight	Relative weight
CW^I^_1_	I	4.351	4.322	4.306	1.004	4.366	0.098
CW^I^_2_	I	4.353	4.286	4.21	1.018	4.431	0.1
CW^I^_3_	I	4.249	4.016	3.875	1.036	4.403	0.099
CW^I^_4_	I	3.982	3.961	3.956	1.001	3.987	0.09
CW^I^_5_	I	4.47	4.335	4.262	1.017	4.546	0.102
CW^I^_6_	I	4.41	4.374	4.369	1.001	4.416	0.099
CW^I^_7_	I	4.362	4.208	3.958	1.063	4.637	0.104
CW^I^_8_	I	4.566	4.535	4.501	1.008	4.6	0.104
CW^I^_9_	I	4.34	4.145	4.096	1.012	4.393	0.099
CW^I^_10_	I	4.501	4.288	4.197	1.022	4.599	0.104
CW^II^_1_	II	4.317	4.304	4.27	1.008	4.351	0.097
CW^II^_2_	II	4.286	4.249	4.197	1.012	4.339	0.097
CW^II^_3_	II	4.501	4.431	4.281	1.035	4.66	0.104
CW^II^_4_	II	4.514	4.486	4.413	1.016	4.589	0.103
CW^II^_5_	II	4.551	4.416	4.301	1.027	4.672	0.105
CW^II^_6_	II	4.532	4.494	4.46	1.008	4.567	0.102
CW^II^_7_	II	4.174	4.122	4.088	1.008	4.209	0.094
CW^II^_8_	II	4.216	4.153	4.06	1.023	4.313	0.097
CW^II^_9_	II	4.174	4.151	4.068	1.02	4.259	0.095
CW^II^_10_	II	4.54	4.509	4.33	1.041	4.728	0.106
CW^III^_1_	III	4.153	4.018	3.842	1.046	4.344	0.099
CW^III^_2_	III	4.545	4.47	4.423	1.011	4.593	0.104
CW^III^_3_	III	4.109	4.07	4.044	1.006	4.135	0.094
CW^III^_4_	III	4.083	4.052	3.953	1.025	4.185	0.095
CW^III^_5_	III	4.514	4.462	4.41	1.012	4.567	0.104
CW^III^_6_	III	4.343	4.306	4.255	1.012	4.396	0.1
CW^III^_7_	III	4.114	4.068	4.034	1.008	4.149	0.094
CW^III^_8_	III	4.527	4.405	4.33	1.017	4.606	0.105
CW^III^_9_	III	4.301	4.195	4.122	1.018	4.377	0.099
CW^III^_10_	III	4.605	4.496	4.444	1.012	4.659	0.106
CW^IV^_1_	IV	4.369	4.309	4.262	1.011	4.417	0.1
CW^IV^_2_	IV	4.358	4.234	4.169	1.016	4.426	0.1
CW^IV^_3_	IV	4.499	4.501	4.434	1.015	4.567	0.103
CW^IV^_4_	IV	4.436	4.382	4.322	1.014	4.498	0.102
CW^IV^_5_	IV	4.239	4.182	4.088	1.023	4.336	0.098
CW^IV^_6_	IV	4.475	4.429	4.369	1.014	4.537	0.102
CW^IV^_7_	IV	4.205	4.156	4.091	1.016	4.272	0.096
CW^IV^_8_	IV	4.275	4.249	4.19	1.014	4.336	0.098
CW^IV^_9_	IV	4.504	4.475	4.418	1.013	4.562	0.103
CW^IV^_10_	IV	4.242	4.135	4.042	1.023	4.34	0.098
CW^V^_1_	V	4.47	4.41	4.369	1.01	4.513	0.102
CW^V^_2_	V	4.145	4.125	4.07	1.013	4.201	0.095
CW^V^_3_	V	4.322	4.312	4.306	1.001	4.327	0.098
CW^V^_4_	V	4.39	4.34	4.2	1.033	4.536	0.103
CW^V^_5_	V	4.299	4.252	4.192	1.014	4.36	0.099
CW^V^_6_	V	4.312	4.273	4.182	1.022	4.405	0.1
CW^V^_7_	V	4.46	4.442	4.384	1.013	4.518	0.102
CW^V^_8_	V	4.309	4.236	4.21	1.006	4.336	0.098
CW^V^_9_	V	4.509	4.483	4.429	1.012	4.565	0.103
CW^V^_10_	V	4.309	4.262	4.223	1.009	4.349	0.099

### Implementation of SERVQUAL

Table 4 shows the values obtained for patient expectations and perceptions of each patient’s needs. The results indicate that in sections I to V, the highest gaps between patient perception and expectation are found for the following needs: “Possibility of presenting a patient’s health status to their caregivers at any time,” “Having professional practitioners and nurses in all working shifts,” “Patient privacy,” “Flexible and open space for activities,” and “Good behavior of practitioners and nurses with children,” respectively.

### Final Weight of Patients’ Needs

The final weights of patients’ needs are shown in Table 4. The results indicate that in sections I to V, the highest weights are assigned to the following needs: “Possibility of presenting a patient’s health status to their caregivers at any time,” “Having professional practitioners and nurses in all working shifts,” “Special attention to patients with no consciousness,” “The safety of devices and equipment,” and “Special attention to children’s health,” respectively.

### Identification of Technical Requirements

**Figure 3.**
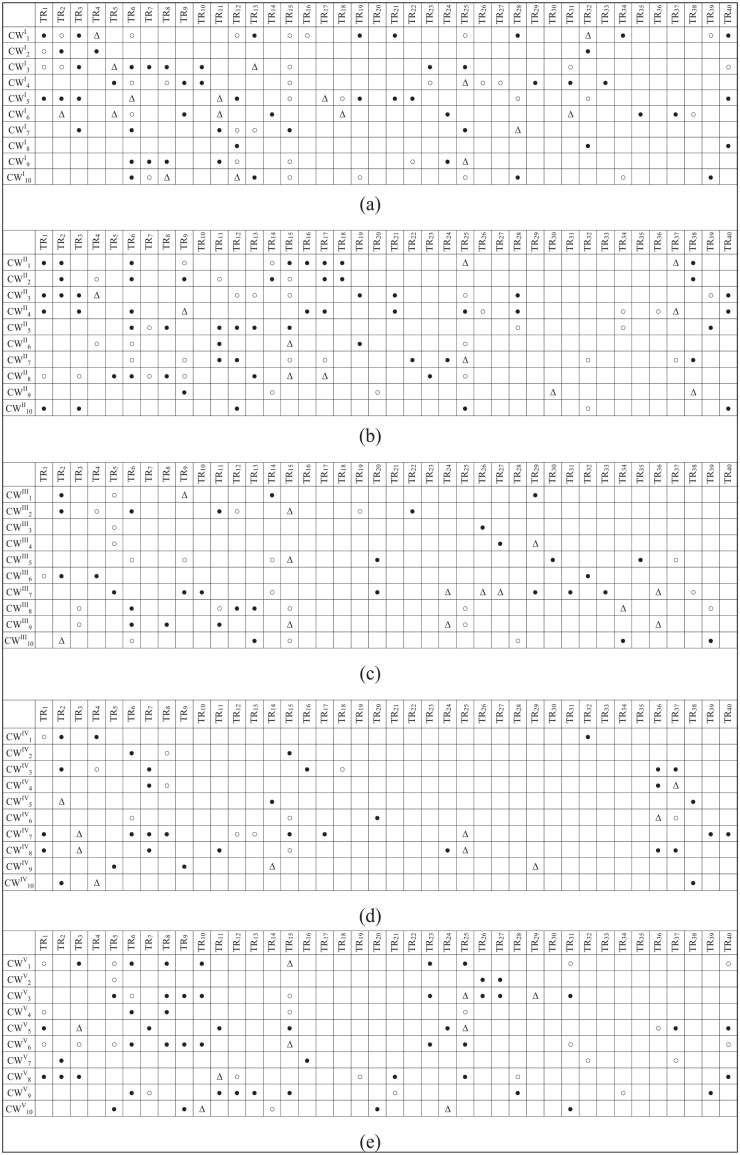
The relationship matrix in various sections: (a) surgery, (b) emergency, (c) CCU, (d) physiotherapy, and (e) pediatric.

In this step, 40 technical requirements were identified to satisfy the patients’ needs. Table 5 shows the identified technical requirements. As shown in Figure 3, each technical requirement addresses at least 2 hospital sections.

**Table 5.** The common technical requirements and their notations.

Notation	Technical requirement	Notation	Technical requirement
TR_1_	Increasing the knowledge of practitioners and nurses	TR_21_	Determining appropriate tests and reducing the time required to obtain the test results
TR_2_	Providing modern medical equipment and facilities	TR_22_	Establish an elaborate admission system and expedite patient clearance procedures
TR_3_	Using experienced specialists, physicians, and surgeons	TR_23_	Taking appropriate measures to increase patient visiting times
TR_4_	Periodic replacement of medical equipment	TR_24_	Using guideboards for guiding visitors
TR_5_	Providing amenities for visitors	TR_25_	Employing experienced nurses
TR_6_	Training practitioners and nurses	TR_26_	Providing high-quality and healthy food
TR_7_	Counseling and instructing the visitors	TR_27_	Providing various types of food for each meal
TR_8_	Taking with patients	TR_28_	Performing appropriate and timely examinations of patients
TR_9_	Improving environmental conditions	TR_29_	Creating personalization options for patients
TR_10_	Using colors and other elements to make the environment of the hospital similar to the home environment	TR_30_	Improving sanitary facilities and equipment
TR_11_	Creating a complete and accurate information system	TR_31_	Providing entertainment for visitors
TR_12_	Estimate the number of patients and eliminate the shortage of workforce	TR_32_	Updating the manager’s knowledge of medical science and management
TR_13_	Determining time periods for checking the patients	TR_33_	Prepare comfortable and clean clothes for patients
TR_14_	Using modern and appropriate structural equipment	TR_34_	Using medical alert devices to monitor patients’ health status
TR_15_	Enhancing the employee’s sense of responsibility	TR_35_	Mechanization of sanitary equipment
TR_16_	Safety equipment for patients’ health	TR_36_	Placing guides and descriptions next to devices and equipment
TR_17_	Create special paths and equipment for patients without walking ability	TR_37_	Placing warning signs
TR_18_	Using modern equipment for patient transfer	TR_38_	Sufficient space for footway
TR_19_	Eliminate the lack of medicines and medical supplies	TR_39_	Increase patient care according to the patients’ age and physical condition
TR_20_	Implementing programs for cleaning the hospital’s environment and training orderlies	TR_40_	Employing specialized physicians for each section

### Obtaining the Relationships Matrix

The relationships between the visitors’ needs and the technical requirements were determined using the fourth questionnaire. The results, presented in Figure 3, indicate that 17 technical requirements cover 5 sections, 12 technical requirements cover 4 sections, 8 technical requirements cover 3 sections, and 3 technical requirements cover 2 sections.

### Weights of Technical Requirements

Using equations (8) and (9), the final and absolute weights for each technical requirement were calculated, and the results are shown in Table 6. The results indicate that the technical requirements with the highest weights are “Training practitioners and nurses (TR6),” “Providing modern medical equipment and facilities (TR2),” “Using experienced specialists, physicians, and surgeons,” and “Enhancing employees' sense of responsibility,” respectively.

**Table 6.** The weights of the technical requirements.

Technical requirement *j*	Relative weights in each section					Relative total weight $(RTW_{j}$ )	Implementation cost or *C_j_* (USD)	Need workforce or *H_j_* (Man-Hour)
	Surgery	Emergency	CCU	Physiotherapy	Pediatric			
TR_1_	0.0398	0.0647	0.0083	0.0606	0.0503	0.0446	10 000	1200
TR_2_	0.0415	0.0437	0.0781	0.083	0.0338	0.0483	1 120 000	2000
TR_3_	0.0602	0.0504	0.0169	0.0057	0.0412	0.0477	230 000	1600
TR_4_	0.0165	0.0114	0.0334	0.0386	0	0.0172	450 000	2000
TR_5_	0.0167	0.0141	0.0472	0.0274	0.0498	0.023	30 000	2000
TR_6_	0.0764	0.0824	0.0939	0.0614	0.0743	0.0788	10 000	800
TR_7_	0.0346	0.0098	0	0.1062	0.0225	0.0279	30 000	800
TR_8_	0.0357	0.0294	0.0247	0.0436	0.0679	0.036	10 000	800
TR_9_	0.0282	0.0438	0.0347	0.0274	0.05	0.0346	20 000	2000
TR_10_	0.0281	0	0.0234	0	0.0524	0.0207	10 000	800
TR_11_	0.0336	0.0487	0.0593	0.0261	0.0359	0.0401	30 000	2000
TR_12_	0.0473	0.0496	0.0346	0.0086	0.0229	0.042	50 000	1200
TR_13_	0.0369	0.0345	0.0523	0.0086	0.0174	0.0347	10 000	800
TR_14_	0.0148	0.0236	0.0409	0.0291	0.0055	0.0202	450 000	1200
TR_15_	0.0449	0.0471	0.0259	0.0701	0.0491	0.0452	30 000	800
TR_16_	0.0049	0.0292	0	0.0275	0.0173	0.0129	80 000	800
TR_17_	0.0017	0.0496	0	0.0257	0	0.0149	30 000	1600
TR_18_	0.0067	0.0284	0	0.0092	0	0.011	20 000	2000
TR_19_	0.035	0.0302	0.0086	0	0.0055	0.0261	30 000	2000
TR_20_	0	0.0046	0.0492	0.0273	0.0166	0.01	10 000	800
TR_21_	0.0299	0.0303	0	0	0.0224	0.024	10 000	2000
TR_22_	0.0202	0.0138	0.0259	0	0	0.0163	10 000	1600
TR_23_	0.0192	0.0141	0	0	0.0506	0.0171	30 000	1200
TR_24_	0.0295	0.0138	0.0053	0.0261	0.0185	0.0216	10 000	800
TR_25_	0.0434	0.0484	0.0169	0.0057	0.0601	0.0406	50 000	800
TR_26_	0.0045	0.005	0.0259	0	0.0326	0.0091	30 000	400
TR_27_	0.0045	0	0.0262	0	0.0326	0.0079	30 000	800
TR_28_	0.0369	0.0353	0.0088	0	0.0229	0.0297	30 000	800
TR_29_	0.0134	0	0.0505	0.003	0.0018	0.0127	30 000	800
TR_30_	0	0.0015	0.0258	0	0	0.0034	80 000	1600
TR_31_	0.0199	0	0.0234	0	0.0445	0.0161	30 000	800
TR_32_	0.037	0.0097	0.0248	0.0265	0.0058	0.0255	30 000	800
TR_33_	0.0134	0	0.0234	0	0	0.0092	30 000	800
TR_34_	0.0198	0.0101	0.0292	0	0.0058	0.016	50 000	800
TR_35_	0.0148	0	0.0258	0	0	0.0102	30 000	1600
TR_36_	0	0.005	0.0053	0.0836	0.0055	0.0076	10 000	800
TR_37_	0.0148	0.0078	0.0086	0.0656	0.0224	0.0162	10 000	800
TR_38_	0.0049	0.0437	0.0078	0.0522	0	0.0176	30 000	2000
TR_39_	0.0203	0.0204	0.0349	0.0257	0.0174	0.0221	30 000	1200
TR_40_	0.0502	0.0457	0	0.0257	0.0446	0.0412	30 000	800

### Resource Constraints

After in-depth interviews with experts, the costs and required workforce for implementing each technical requirement are estimated. Table 6 shows the estimated costs and required workforce for each technical requirement. Based on the total relative weight and the resource constraints, the most suitable technical requirements for implementation, considering the budget limit, can be selected using the mathematical model (equations (10)-(12)). In equation (10), $RTW_{j=1,\ldots ,40}$ =[0.0446,0.0483,0.0477,. . .,0.0412] represents the vector of coefficients of $X_{j=1,\ldots ,40}$ for the case study, which is the total relative weight of each technical requirement. In equation (11), $C_{j=1,\ldots ,40}$ =[10 000, 1 120 000, 230 000,. . ., 30 000] represents the implementation cost of each technical requirement in the case study hospital. In equation (12), $H_{j=1,\ldots ,40}$ =[1600, 2000,. . ., 800] represents the required workforce (man-hours) for each technical requirement in the considered hospital.

Suppose the total available budget equals the sum of all technical requirements’ costs (3 250 000 USD) and the available workforce equals the sum of all needed workforce (48 400 man-hours). In that case, all the technical requirements would be selected for implementation, and the benefit obtained for the hospital would be 1 (the sum of the weights of all technical requirements). However, when the budget or available workforce is limited, not all requirements can be selected, necessitating an optimal selection of technical requirements. This optimization problem is addressed in this study by solving equations (10)-(12) using a commercial optimization solver (LINGO 18 software).

Table 7 presents the results obtained for different budget and workforce values. As shown in this table, reducing the budget while keeping the available workforce constant decreases the number of selected technical requirements and, consequently, the value of the objective function (Scenarios 1-5). Similarly, this effect is observed when reducing the available workforce while keeping the budget constant (Scenarios 6 to 10).

**Table 7.** The results obtained for different budgets and available workforce.

Scenario	Considered budget (USD)	Available workforce (man-hour)	Selected technical requirements	Objective function
0	*B* = 3 250 000	AWF = 48 400	All technical requirements	1
1	0.9×B	AWF	All technical requirements except TR4	0.9828054
2	0.7×B	AWF	All technical requirements except TR4, TR14, and TR30	0.9592515
3	0.5×B	AWF	All technical requirements except TR2, TR4, and TR30	0.9311335
4	0.3×B	AWF	All technical requirements except TR2, TR4, TR14, TR16, TR26, TR27, TR30, and TR34	0.8649875
5	0.1×B	AWF	TR6 to TR13, TR15, TR20 to TR22, TR24, TR28, and TR37	0.5635771
6	B	0.9× AWF	All technical requirements except TR18, TR30, and TR35	0.9754287
7	B	0.7× AWF	All technical requirements except TR4, TR17, TR18, TR22, TR27, TR30, TR33, TR35, TR36, and TR38	0.8846869
8	B	0.5× AWF	TR1, TR2, TR3, TR6, TR7, TR8, TR9, TR10, TR11, TR12, TR13, TR14, TR15, TR24, TR25, TR26, TR28, TR31, TR32, TR34, TR37, TR39, TR40	0.7589405
9	B	0.3× AWF	TR1, TR2, TR3, TR6, TR7, TR8, TR12, TR13, TR15, TR24, TR25, TR26, TR28, TR32, TR40	0.5728919
10	B	0.1× AWF	TR6, TR8, TR13, TR15, TR25, TR40	0.2765436

### Discussion

#### Discussion of Results

The analysis of the MD-HOQ results reveals that the technical requirements with the highest priorities are “Training practitioners and nurses (TR6),” “Providing modern medical equipment and facilities (TR2),” and “Using experienced specialists, physicians, and surgeons (TR3).” Each requirement plays a vital role in enhancing hospital quality and services.

Training practitioners and nurses is crucial to enhance hospital performance across various dimensions. It improves patient care by equipping healthcare professionals with updated knowledge of medical advancements and new technologies.^47-49^ Furthermore, comprehensive training reduces errors by reinforcing adherence to protocols and safety measures, thereby increasing patient safety.^47-50^ Effective training programs also enhance communication skills, fostering better patient interactions characterized by empathy and understanding.^47,48,50^ From an operational standpoint, familiarity with standardized practices and efficient use of equipment increases overall efficiency,^47,51^ while compliance with legal and regulatory requirements is strengthened through informed training initiatives.^6,51^ In addition to these benefits, professional development opportunities significantly boost job satisfaction and staff retention, reducing turnover and ensuring a consistent, skilled workforce.^51,52^ Collectively, these factors contribute to higher patient satisfaction, which positively impacts clinical outcomes.^47^

The provision of modern medical equipment and facilities also significantly improves hospital services. Advanced diagnostic tools enhance the accuracy of diagnoses, enabling better treatment planning and patient outcomes.^53-55^ Similarly, cutting-edge equipment facilitates the execution of complex treatments and surgeries with greater precision and safety.^53-58^ At the same time, real-time monitoring devices reduce the likelihood of critical incidents, thus enhancing patient safety.^54,57,58^ Modernization extends beyond patient care; automated systems streamline hospital operations, reducing waiting times and optimizing resource utilization.^53,56-59^ Hospitals with state-of-the-art facilities also attract skilled medical professionals who prefer to work in advanced environments.^54,56^ Furthermore, these technologies enable continuous care, including remote monitoring post-discharge,^53-58^ and reduce costs by minimizing errors and complications.^53-55,59^ Compliance with global standards is another important outcome, as modern equipment enhances a hospital’s reputation and credibility, drawing more patients and ensuring competitive positioning.^53,55^

Similarly, employing experienced physicians and surgeons significantly impacts hospital quality. Their expertise leads to higher survival rates, fewer medical errors, and better treatment outcomes.^60,61^ These specialists also contribute to operational efficiency by enabling faster and more accurate diagnoses, which reduce hospital stays and associated costs.^61,62^ Beyond technical proficiency, experienced practitioners excel in patient communication, fostering trust and satisfaction through clear and empathetic interactions.^62,63^ Their credibility encourages patients to adhere to treatment plans, improving outcomes.^63,64^ Additionally, these experts serve as mentors, transferring knowledge to junior staff and strengthening the overall competency of the medical team.^63^ Hospitals that employ experienced specialists often enjoy enhanced reputations, which not only attract more patients but also facilitate access to financial resources for further development.^64^

#### Discussion of the Techniques Used

The hybrid methodology employed in this research integrates SERVQUAL, MD-HOQ, and a mathematical model to optimize hospital resource allocation and prioritize technical requirements effectively.

SERVQUAL plays a central role by combining the importance of patient needs with their dissatisfaction levels, as measured by the gap between expectations and perceptions. For example, the need for the “Possibility of presenting a patient’s health status to their caregivers at any time” (CW7) has a lower importance degree (4.362) compared to “Adequate staff availability” (CWI8, with a value of 4.566), its relative weight (4.637) is higher because patients exhibit greater dissatisfaction with CW7 (improvement ratio of 1.063) than with CWI8 (improvement ratio of 1.008). This approach ensures that the most critical needs, from the patient’s perspective, are prioritized.

MD-HOQ enhances the coordination of quality improvement efforts across hospital sections by integrating the priorities of different units into a unified framework. Without this integration, separate implementations of HOQ could result in inefficiencies, such as resource misallocation or rework. For instance, in the physiotherapy section, the technical requirement “Counseling and instructing visitors” (TR7) has the highest priority with a relative weight of 0.1062. However, when considering the overall hospital context, its weight decreases to 0.0279, demonstrating the importance of aligning section-specific priorities with organizational goals. Moreover, MD-HOQ incorporates the relative importance of hospital sections (determined through AHP), ensuring that resources are allocated to maximize benefits for the entire organization.

The mathematical model further refines this prioritization by accounting for resource constraints, such as costs and workforce availability. Unlike traditional approaches that prioritize requirements solely based on their weights, this model optimizes utility by selecting a combination of requirements that delivers the highest overall value within the available resources. For instance, in Scenario 10 (Table 7), where 4840 (0.1 × AWF = 0.1 × 48 400) human resources are available, selecting requirements based solely on weight would result in implementing only TR6, TR2, and TR3, consuming all resources and achieving a total weight of 0.1748. By contrast, the mathematical model selects TR6, TR8, TR13, TR15, TR25, and TR40, achieving a higher total weight of 0.2765. This demonstrates how the model effectively increases utility by balancing priority, cost, and resource constraints.

## Conclusion and Future Research Directions

This study presented an integrated Quality Function Deployment (QFD) approach with an overarching view, utilizing a new Multi-Dimensional House of Quality (MD-HOQ) matrix for quality improvement in hospital services. Additionally, MD-HOQ was integrated with Service Quality (SERVQUAL) analysis. This proposed approach helps hospital managers prioritize technical requirements more effectively. Since implementing each technical requirement incurs costs and requires workforce, correct prioritization considering resource constraints leads to higher patient satisfaction. Integrating SERVQUAL analysis into the traditional HOQ introduces an additional dimension in determining the weight of technical requirements. This approach considers the gap between patients’ expectations and perceptions of each need. Since a larger gap indicates greater patient dissatisfaction, technical requirements that address needs with higher gaps receive higher implementation priority. Moreover, hospitals consist of different sections, and MD-HOQ provides a tool to compile a comprehensive list of technical requirements for all hospital sections. In the proposed MD-HOQ, the weight of each technical requirement is determined based on its effects on patient needs across all hospital sections. This approach ensures that hospital resources are allocated to the most impactful technical requirements, benefiting the entire hospital.

To test the proposed approach, it was applied to a hospital. First, hospital sections and their importance were identified. Patients’ needs in various hospital sections were then identified, and their importance degree, expectations, and perceptions were obtained. Consequently, a list of technical requirements to satisfy these patient needs was identified, and the weight of each technical requirement was calculated based on its impact on patient needs. Finally, a mathematical formulation was used to determine the optimal list of technical requirements, considering resource constraints such as budget and available workforce.

By focusing on enhancing hospital services and balancing quality with resource constraints, this study contributes valuable insights into quality management, hospital services, and the application of MD-HOQ frameworks integrated with SERVQUAL analysis. This approach not only improves the overall quality of hospital services but also ensures efficient utilization of resources, ultimately leading to better patient outcomes and satisfaction.

### Research Implications

This research provides hospital managers with a comprehensive understanding of patients’ needs across different sections. It also facilitates knowledge sharing, coordination, and cooperation between hospital sections. By offering an integrated plan to address patients’ needs in multiple sections simultaneously, this study promotes better utilization of hospital resources, eliminates rework, saves time, and increases hospital productivity. Additionally, integrating MD-HOQ and SERVQUAL enhances hospital resource use and maximizes hospital service quality while considering resource constraints. Ultimately, improving the quality of services in hospitals positively influences society’s overall health.

### Research Limitations and Suggestions for Future Studies

The results obtained from the questionnaires are specific to the case study considered and may not be applicable to other cases with different needs and specifications. However, the proposed approach offers potential for application to other organizations with multiple sections and distinct types of customers. Additionally, the patient needs and technical requirements identified in this study may be a starting point for similar studies in other hospitals.

Future research could explore using other MCDM techniques, such as the analytic network process (ANP) or the technique for order of preference by similarity to ideal solution (TOPSIS), to determine the weight of patient needs or sections. Another potential research avenue is incorporating fuzzy numbers to account for uncertainty in parameters such as the importance, perception, and expectations of patient needs. Furthermore, using other quality management tools, such as the Kano model, to determine the weight of patient needs could be considered as another area for future research.

## Statements and Declarations

**Ethical Considerations:** This study involved the completion of anonymized questionnaires. Ethical approval was not required as the study did not involve personal data, medical interventions, or invasive procedures. The research strictly adhered to the principles outlined in the Declaration of Helsinki. Informed consent was not required for this study as per the “Code of Ethics in Medical Research of the Islamic Republic of Iran” (adopted by the National Committee for Ethics in Biomedical Research, 2019). This is because the data collected were fully anonymized and did not include personal identifiers such as names, contact information, or other sensitive details. The anonymization process ensured that the data could not be traced back to individual participants, thereby fully safeguarding their privacy. As a result, the study adhered to ethical standards without the need for explicit informed consent.

**Data Availability:** The data used or analyzed in this study are available from the corresponding authors upon reasonable request.
